# Biomine: predicting links between biological entities using network models of heterogeneous databases

**DOI:** 10.1186/1471-2105-13-119

**Published:** 2012-06-06

**Authors:** Lauri Eronen, Hannu Toivonen

**Affiliations:** 1 Biocomputing Platforms Ltd, Innopoli 2, Tekniikantie 14, , FI-02150 Espoo, Finland; 2 Department of Computer Science and HIIT, University of Helsinki, PO Box 68, Helsinki, FI–00014, Finland

## Abstract

**Background:**

Biological databases contain large amounts of data concerning the functions and associations of genes and proteins. Integration of data from several such databases into a single repository can aid the discovery of previously unknown connections spanning multiple types of relationships and databases.

**Results:**

Biomine is a system that integrates cross-references from several biological databases into a graph model with multiple types of edges, such as protein interactions, gene-disease associations and gene ontology annotations. Edges are weighted based on their type, reliability, and informativeness. We present Biomine and evaluate its performance in link prediction, where the goal is to predict pairs of nodes that will be connected in the future, based on current data. In particular, we formulate protein interaction prediction and disease gene prioritization tasks as instances of link prediction. The predictions are based on a proximity measure computed on the integrated graph. We consider and experiment with several such measures, and perform a parameter optimization procedure where different edge types are weighted to optimize link prediction accuracy. We also propose a novel method for disease-gene prioritization, defined as finding a subset of candidate genes that cluster together in the graph. We experimentally evaluate Biomine by predicting future annotations in the source databases and prioritizing lists of putative disease genes.

**Conclusions:**

The experimental results show that Biomine has strong potential for predicting links when a set of selected candidate links is available. The predictions obtained using the entire Biomine dataset are shown to clearly outperform ones obtained using any single source of data alone, when different types of links are suitably weighted. In the gene prioritization task, an established reference set of disease-associated genes is useful, but the results show that under favorable conditions, Biomine can also perform well when no such information is available.

The Biomine system is a proof of concept. Its current version contains 1.1 million entities and 8.1 million relations between them, with focus on human genetics. Some of its functionalities are available in a public query interface at http://biomine.cs.helsinki.fi, allowing searching for and visualizing connections between given biological entities.

## Background

Biological databases contain a vast amount of readily accessible data concerning the function and relationships of genes and proteins, such as protein interactions, genes’ effects on diseases and functional gene annotations. Here, we introduce Biomine, a system that integrates data from several such databases under a common graph data model and repository, with the goal of enabling discovery and evaluation of connections spanning multiple types of relationships derived from different source databases. Such indirect relationships can act as hypotheses for potential, yet undiscovered links, or they can be used to describe and validate relationships obtained from experimental data.

**Figure 1:**
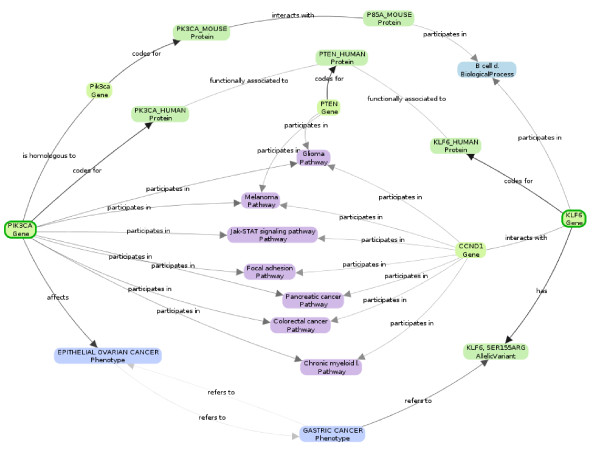
**A subgraph summarizing the relationships between 2 genes related to gastric cancer.** The query genes, PIK3CA and KLF6, are marked with a green border. The other nodes in the graph are the ones on the strongest paths linking the query genes, as defined by the edge-weighting scheme described later in this article.

Biomine represents the knowledge extracted from the source databases using an abstract and efficiently accessible graph representation: biological entities and concepts (the nodes) linked by their known relationships (the edges). Figure 1 shows a small example graph illustrating the contents of the database.

A practical motivation for our work is prioritization of putative disease genes resulting from genome-wide association studies [1]. Such studies typically produce a large number of genes showing statistical association with the disease in question, of which only a fraction are actually biologically related to the disease. An important task is to identify the actually relevant genes from this list of putative disease genes.

The main idea underlying the work in this paper is that genes and their protein products do not function in isolation, but rather as part of a large network of molecular interactions. Thus the impact of a specific genetic abnormality is not restricted to the gene product that carries it, but can spread along the links of the network and alter the activity of functionally related gene products. Consequently, mutations in functionally related genes (e.g. participating in the same pathway or protein complex) will often affect the same diseases [2]. Analysis of disease-related protein networks has shown that proteins involved in a disease tend to physically interact with other proteins involved in the same disease [3]. With the recent rise in the availability of molecular network data, it has become practical to predict disease-affecting genes based on the network of biological relationships (see [4-6] for reviews).

As another example application, consider protein interaction measurements from genome-wide protein interaction screens [7]. A major issue with this kind of data is the prevalence of spurious interactions. An analysis by Deane et al. [8] suggests that only 30-50 % of interactions derived by such methods are biologically relevant. The problem of separating the true interactions from spurious ones can be formulated as a link prediction problem within the graph of already known interactions.

We formulate the protein interaction prediction and gene prioritization tasks as instances of link prediction, where the goal is to recognize pairs of nodes that should be (or will be) connected by an edge. The predictions are based on a proximity measure computed on the integrated graph. We consider and evaluate a number of proximity measures that are based on weights assigned to the individual graph edges. These weights are based on three factors: the type of the relationship, an informativeness measure based on the number of other nodes linked to each node, and reliability or other suitable edge-specific score available in some of the source databases. We evaluate the power of this approach for prediction in a challenging setting: we use proximities of nodes in a 3-year-old version of the Biomine database to predict the appearance of new links to the current database version.

The contributions that follow in subsequent sections can be summarized as follows.

1.We introduce Biomine, an integrated network of biological entities from heterogeneous source databases.

2.We systematically evaluate Biomine in two challenging link discovery settings: protein interaction and gene–phenotype relationship prediction. The evaluations consist of comparing several node proximity measures, assessing the importance of different data sources for the link prediction task, and optimizing the weights of different types of links.

3.We show how to apply Biomine to the task of disease gene prioritization, and propose a new clustering-based gene prioritization method which is applicable when there is no pre-existing reference set of known disease genes available.

An early version of Biomine has been outlined by Sevon et al. [9]. To make this article self-contained, we provide an updated description of the edge weighting method introduced by Sevon et al.

### Related work

We will next briefly review representative related work in the areas of integrating biological networks, graph-based disease gene prediction, and general node proximity measures for link prediction.

#### Integrating biological networks

Data about relationships of biological entities is readily available in numerous public databases. To enable joint analysis of such data, the data needs to be integrated and made accessible under a uniform query interface. Several such data integration systems have been proposed in the literature. Of these, most similar to our approach are ONDEX [10] and Biozon [11], which both collect the data from various sources under a single data store, using a graph data schema centered around the non-redundant set of biological objects shared by each data source. The data model in both systems is a graph with typed nodes and edges, allowing for the incorporation of arbitrary data sources. In addition to curated data derived from the source databases, both ONDEX and Biozon include in-house data such as similarity links computed from sequence similarity of proteins and predicted links derived by text mining. Biozon provides several types of queries, e.g. searching by graph topology and ranking of nodes by importance defined by the graph structure. In ONDEX, the integrated data is accessed by providing a standard pipeline, in which individual filtering and graph layout operations may be combined to process the graph in application-specific ways. BioWarehouse [12] aims to provide generic tools for enabling users to build their own combinations of biological data sources. Their data management approach is rather similar to ONDEX and Biozon, but the data is stored in a relational database with a dedicated table for each data type instead of a generic graph structure. This approach allows database access through standard SQL queries. Biomart [13] tackles the problem not by collecting data into a central location, but instead by representing the data in each original source database using a data storage service with a standard format, enabling them to be accessed through a uniform web-based query interface.

### Graph-based disease gene prediction

A simple approach for predicting potential disease genes is to just assign interaction partners of already known disease-related proteins as potential candidates [14]. Krauthammer et al. [15] use a more elaborate method, where evidence from known disease genes is propagated to nearby candidate genes according to a score based on shortest paths distance. Kohler et al. [16] also use a related approach, based on a random-walk-based network proximity measure instead of considering only a direct neighborhood or shortest paths. Vanunu et al. [17] take an even more global view, and expand the query from the given disease to include other diseases with phenotypic similarity. Evidence is then propagated in a protein interaction network from all proteins known to be related to any of these diseases.

In contrast to the previous methods, which only use protein interaction data, Franke et al. [18] and Linghu et al. [19] both construct a network of functional associations (“functional linkage network”) using multiple types of integrated source data, and use mutual proximities of genes in this graph as supporting evidence for the disease association. They construct a network of functional associations using machine learning techniques to combine evidence from different data sources, using a fixed cutoff value to remove unreliable associations. Franke et al. [18] evaluate each candidate gene based on the shortest path distance to other candidate genes, while Linghu et al. [19] only use information from neighboring genes.

Hwang and Kuang [20] consider both multiple types of associations (edges) and multiple types of nodes. Instead of integrating them all together into a homogeneous network, they propose methods to propagate information in the network while taking its heterogeneity into account.

An alternative to network-based approaches is to utilize pre-known sets of functionally related genes [21,22]. The idea in these methods is that each known pathway (or other pre-defined set of functionally related genes) is tested for relative excess of disease-associated genes, accumulating information from several modest associations into a single, stronger signal. The results of Chasman [22] indicate that while extremely strong associations remain best identified by conventional methods, the gene set approach provides a useful complementary mode of analysis for revealing modestly associated genes for complex diseases.

Literature mining is a popular source of information for hypothesis generation in biological discovery [23]. Hristovski et al. [24] predict disease genes using co-occurrence statistics of terms. Following Swanson’s ideas [25], they look for strong connections of exactly two hops in a network of term co-occurrences: if the given disease name *X* co-occurs with some terms *Y * which in turn co-occur with some genes *Z*, then *Z* are candidate genes for disease *X*. Hristovski et al. additionally take into account chromosomal location.

A practical overview of freely available web tools to prioritize candidate genes is provided by Tranchevent et al. [26].

### Node proximity measures for link prediction

Many node proximity measures have been proposed for link prediction in unweighted graphs. For an experimental comparison of these measures, see Liben-Nowell and Kleinberg [27]. For the weighted graphs considered here, much less has been published.

Asthana et al. [28] were the first to use network reliability for link prediction in protein networks. In our earlier publication [9], we used two alternative proximity measures: probability of best path, and network reliability measured in a subgraph consisting of a fixed number of best paths. Potamias et al. [29] recently introduced expected-reliable-distance for link prediction in probabilistic weighted graphs. Random walk methods are a popular choice for measuring proximity in networks (see, e.g., [16,29,30]), and can be straightforwardly extended to work with weighted graphs. We will review the exact definitions of the above-mentioned measures later.

Hwang and Kuang [20] and Vanunu et al. [17] propose different propagation methods for measuring proximity of nodes that are not directly connected. Like random walk and network reliability, their measures are global in the sense that they consider — at least in principle — connectivity using the whole network. These methods seem to assume structured information about phenotype similarity and known disease genes.

The field of network-based protein function prediction (see, e.g., [31] for a review) is also related to the work in this study, and many methods have been developed to annotate proteins of unknown function based on existing annotations and network data. However, these methods are not directly applicable to the problem of predicting arbitrary edges in weighted graphs.

## Methods

We next describe the Biomine database, and then give node proximity measures that can be used for link prediction. (Disease gene prioritization methods are deferred to the Results section, to be presented in the context of that particular application.)

### The Biomine database

The Biomine graph database essentially is an integrated index of several biological databases, each with different contents and format. Biomine has a relatively simple data model: a labeled graph with typed nodes and edges. Distinct entities of the source databases, such as genes, proteins and gene ontology (GO) concepts, are the nodes of the Biomine database, and connections (cross-references) between entities, such as GO annotations, gene-protein relationships and protein interactions, are edges between nodes. Additionally, nodes and edges can have arbitrary attributes, such as names and reliabilities, to represent additional data from the source databases. In this section, we describe the database contents and data model, including the edge-weighting scheme.

#### Database contents

We first briefly review the source databases indexed by Biomine and summarize what data is derived from each of them. The contents of Biomine are summarized in Tables 1 and 2. Currently, data is extracted and stored for human and four model organisms: mouse, rat, fruit fly and nematode (c. elegans). The current primary topic is human biology, and the additional organisms are included to enable predictions based on potentially more comprehensively annotated homologous genes in these model organisms.

**Table 1:** Summary of node types in Biomine

**Node type**	**Source databases**	**Count**	**Mean degree**
Article	PubMed	533,000	3.9
Protein	UniProt, STRING	275,000	29.6
Gene	Entrez Gene	193,000	18.6
Homology group	Entrez HomoloGene	25,000	3.2
Biological process	GO	20,000	32.5
Gene variant	OMIM	19,000	1.4
Gene location (Locus)	Entrez Gene, OMIM	12,000	18.7
Protein family	InterPro	12,000	69.6
Molecular function	GO	10,000	49.1
Drug	KEGG	8,800	0.7
Protein feature	InterPro	8,000	69.6
Phenotype	OMIM	6,500	17.0
Enzyme	KEGG	5,100	10.2
Cellular component	GO	2,900	122.2
Pathway	KEGG, UniProt	1,800	37.1
Tissue	UniProt	1,300	189.1
		total 1,133,000	14.8

**Table 2:** Most important edge types in Biomine

**Edge type**	**Source databases**	**Count**
Protein *is associated to* Protein	STRING	2,916,000
Article *refers to* Node	Entrez Gene, UniProt, KEGG, OMIM	2,250,000
Node *has annotation* GO	Entrez Gene, UniProt, InterPro	1,365,000
Protein *contains* Feature	UniProt	507,000
Gene *is homologous to* Gene	HomoloGene	259,000
Gene *codes for* Protein	Entrez Gene, STRING	174,000
Gene *is located in* Locus	Entrez Gene, OMIM	151,000
Protein *belongs to* Family	UniProt	114,000
Protein *interacts with* Protein	Entrez Gene, UniProt	98,000
OMIM *refers to* OMIM	OMIM	85,000
Gene *participates in* Pathway	KEGG	65,000
GO *has parent* GO	GO	56,000
InterPro *has parent* InterPro	InterPro	20,000
Compound *participates in* Pathway	KEGG	9,700
Gene *affects* Phenotype	Entrez Gene	5,100
Phenotype *is mapped to* Locus	OMIM	3,400
		total 8,078,000

“Node” denotes any type of node, “OMIM” denotes nodes of type “Gene”, “Gene variant” or “Phenotype”, “GO” denotes nodes of type “Molecular function”, “Cellular component” or “Biological process“, and “InterPro” denotes nodes of type “Protein family” or “Protein feature”. Edge types with less than 3,000 instances are not listed.

NCBI’s *Entrez Gene*[32,33] database is the main source of gene annotation in Biomine. The gene nodes in Biomine are derived from the respective gene entries in Entrez. The main types of edges derived from Entrez are gene ontology (GO) annotations, protein interactions, cytogenetic gene locations, and information about genes’ participation in diseases (represented by Phenotype nodes). Additionally, genes are linked to their protein products and homologous genes in other organisms. Homology relationships are derived from another Entrez database, *HomoloGene*[33].

*UniProt*[34] is the main source of protein-related information. Its core elements are proteins, pathways and tissues, mapped to nodes in Biomine. Edges derived from UniProt include GO annotations for proteins, protein interactions, participation of proteins in pathways, membership in protein families and other protein features (defined by the InterPro database, see below), as well as tissue-specificity of proteins.

*Gene Ontology* (GO) aims to provide a controlled vocabulary for genes and gene products [35]. Its core domains are cellular components, biological processes and molecular functions. The ontology is structured as a directed acyclic graph, and each term has defined relationships to one or more other terms in the same domain, and sometimes to other domains. This graph is imported as part of the Biomine graph. The actual annotations linking genes and proteins to GO nodes are derived from other databases, primarily from Entrez Gene and UniProt, as described above.

*InterPro*[36] is a protein signature database that enables classification of proteins into families and functional protein features, which are mapped into nodes in Biomine. The InterPro entries (protein families and features) are organized into a hierarchy, represented as edges between the InterPro nodes. The actual edges linking protein and InterPro nodes are derived from the UniProt database.

*STRING*[37] contains known and predicted functional associations between proteins, based on text mining and sequence analysis, as well as protein-related information from other databases. These are directly mapped to Biomine as edges between proteins. In addition, each association contains a score between 0 and 1 reflecting the confidence in the prediction, which is stored to Biomine as edge reliability (see Subsection *Weighting of edges* below).

*Online Mendelian Inheritance in Man* (OMIM) is a catalogue of human genes and genetic disorders [38], and is the main source of phenotype information in Biomine. There are two types of records in OMIM: genes and phenotypes. The former are mapped to corresponding gene IDs in Entrez and are merged into a single node in Biomine with the OMIM ID as a secondary identifier. Phenotype records are represented in Biomine by dedicated nodes. The OMIM database also contains descriptions of allelic variants and gene locations, mapped to their distinct node types, and a large number of references to biomedical literature. The OMIM records consist of text with references to other OMIM records; these references are mapped to edges in Biomine.

*Kyoto Encyclopedia of Genes and Genomes* (KEGG) is a large, integrated database resource consisting of 16 main databases, broadly categorized into systems information, genomic information, and chemical information [39]. Biomine uses a subset of KEGG, including pathways as the main node type, and the participation of genes, drugs and compounds in each pathway represented as edges between the corresponding node and pathway.

*PubMed*[33] is a freely accessible on-line database of biomedical journal citations and abstracts with approximately 20 million entries at the time of writing. In addition to the data types listed above, many of the source databases (Entrez, UniProt, InterPro and OMIM) contain references to PubMed entries, to represent the fact that the corresponding entity is mentioned in the article. In Biomine, these referenced PubMed entries are represented by Article nodes. From PubMed itself, we only import the titles of these articles.

##### Data management

The data storage is organized as a data warehouse: information from several source databases is first extracted and transformed into a general data format, and subsequently stored in a local relational database (MySQL) for easy access by query programs. This approach is motivated by the requirement to rapidly perform complex queries needed by the link prediction methods, which excludes the online use of services (such as Entrez e-utils). Also, it enables performing some integrity checks of cross-references between the independently maintained data sources. On the other hand, we essentially only store an index: identifiers and additional names of entities, cross references between entities (i.e., edges with weights), and URL’s to the original records in the source databases.

The database is updated periodically with new data from the source databases. During the update process, synonyms, invalid references, and other anomalies are resolved, and the resulting data is compared to the previous version of the database to detect loss of data resulting, e.g., from formatting changes in source databases. The details of the complete conversion and importing process are out of the scope of this paper.

In addition to the relational database, we use a dedicated cache server which stores the graph structure in a compact format in main memory, enabling significantly faster queries than would be possible by using only the relational database. A public query interface to the database is available at http://biomine.cs.helsinki.fi.

#### Data model

We now present the Biomine data model more formally. The Biomine database is a directed, labelled and weighted graph *G* = (*V*,*E*,*p*) where *V * and *E* are the sets of nodes and edges, respectively, and *p*:*E* ↦ [0,1] associates a probability *p*(*e*) to each edge *e* ∈ *E*.

The nodes *v* ∈ *V* are labelled by a type from set *T*_*v*_, such as Gene or Protein. *T*_*v*_ consists of the node types in the left column of Table 1. We denote the node type mapping by *t*_*v*_:*V* ↦ *T*_*v*_.

The set *E* ⊂ *V* × *V* of edges consists of node pairs (*u*,*v*). (Biomine supports parallel edges, but for simplicity we ignore them here. The extension is straightforward.) Edges are weighted. Edges have labels from the edge type set *T*_*e*_; we denote the mapping from edges to their types by *t*_*e*_:*E* ↦ *T*_*e*_. Edge types represent relations between nodes, such as “Gene *codes for* Protein” or “Article *refers to* Gene”. The left column of Table 2 lists the most common edge types in *T*_*e*_.

Each edge type *τ* ∈ *T*_*e*_ has an inverse type *τ*^−1^. For instance, the inverse of “Gene *codes for* Protein” is “Protein *is coded by* Gene”. For each edge *e* = (*u*,*v*) ∈  *E* we assume that its inverse edge (*v*,*u*) with type *t*_*e*_(*e*)^−1^ always exists in *E*. Hence, the graph is effectively undirected but has directed edge types.

In most cases there is only one possible edge type for a pair of node types. For example, an edge between a gene and a protein always has the type *codes for*. In some cases, though, there are different edge types between the same types of nodes, such as experimentally verified protein interaction vs. predicted protein association, or gene homology vs. textual reference between two gene records in OMIM.

#### Weighting of edges

Not all edges in Biomine are equally important. For example, an experimentally verified protein interaction (an edge of the type Protein *interacts with* Protein) should probably have a higher weight than a predicted one (Protein *is functionally associated to* Protein). Similarly, annotated knowledge about a gene’s effect on a disease is probably more important than just knowing that the gene and the disease are mentioned in the same article.

As an example of a second type of edge importances, consider two articles, where one refers to 2 genes and the other one to 20 genes. Since the former article is more specific, the corresponding edges are likely to be more informative.

A third and most obvious case of different importances is when a source database specifies a weight or score for a relation such as the confidence of predicted associations in the STRING database [37].

In Biomine, we formalize the three above-mentioned factors as follows.

1.*Relevance.* Each edge type *τ* has a fixed *relevance coefficientq*_*τ*_ ∈[0,*∞*] representing the relative importance of that relationship type. We denote the relevance of an arbitrary edge *e* by *q*(*e*). The suitable choice of values for each *q*_*τ*_ is ultimately dependent on the specific application at hand. In Subsection *Link prediction methods*, we will experimentally choose the relevances such that they maximize link prediction accuracy. Relevances can also be set manually, to reflect the user’s subjective interests.

2.*Informativeness.* The *informativenessi*(*u*,*v*) ∈[0,1] of an edge (*u*,*v*) is measured based on the degrees of its incident nodes. As a simple method to penalize a node *u* with a high degree *deg*(*u*), we take some negative power *deg*(*u*)^−*α*^ of it. Here 0 ≤ *α* ≤ 1 is a parameter controlling how steeply the informativeness decreases with increasing node degree. The informativeness of an edge (*u*,*v*) is then defined by the degrees of both its endnodes:

$$i(u,v)=\sqrt{\deg{\left(u\right)}^{-\alpha }\cdot \deg{\left(v\right)}^{-\alpha }}.$$

Based on preliminary experiments with different values of *α*, we by default set *α* = 0.25. (Where needed, this parameter can be optimized e.g. by systematically testing different values, possibly in combinations with other parameters. A thorough optimization of all parameters is not within the scope of this paper, however.)We consider two versions of degree penalty. The *linktype-independent* penalty simply uses the ordinary degrees of nodes. The *linktype-specific* penalty only takes the degree with respect to edges of the same type as edge (*u*,*v*).

3.*Reliability.* The *reliability* of an edge *e*, denoted by *r*(*e*) measures how confident we are that the relationship ∈[0,1], measures how confident we are that the relationship represented by the edge actually exists. From the STRING database, we obtain a reliability value for each predicted edge *e*, directly mapped to Biomine as *r*(*e*). For edges derived from other databases, *r*(*e*) is currently defined to be one, as they contain manually curated information which is expected to be reliable.

We combine these three factors into an overall edge weight by simply taking their product:

p(e)=q(e)·i(e)·r(e).

In the next section, we will define general node proximity measures based on the edge weights. The above definition is directly applicable when using random walk as the node proximity measure. However, for probabilistic proximity measures, edge weights need to be in [0, 1], and consequently the following modification is used:

p(e)=min(q(e)·i(e)·r(e),1).

In this case weight *p*(*e*) can be interpreted as the probability that *e* represents an actually existing, relevant and informative relationship.

### Link prediction methods

The computational problem that we consider is *link prediction*, the prediction of relationships that are not obvious in the existing data. The motivation for this is that the graph of biological knowledge is far from complete; most of the information is still missing, but the existing data can potentially help identify some of the missing pieces.

#### Link prediction as node proximity measurement

In the most concrete form, link prediction means predicting the appearance of new edges into the database. We also use the term “link” in a broader sense, however. For example, in the experiments we will predict whether two genes will be discovered to affect the same disease (represented by two Gene *affects* Phenotype edges). As a more complex example, we predict whether a gene affects susceptibility to a disease, based on its proximity to other genes already known to affect the disease.

We base link prediction on a graph-based node proximity measure. The assumption is quite simply that nodes closely connected in the graph are likely to be related also biologically, potentially warranting the addition of a direct link to one of the source databases. (In machine learning, this is known as transductive learning.) Given the huge number of nodes in Biomine, resulting in an even larger number of potentially related node pairs, it is obviously not feasible to predict links without some prior information about the set of potential links. Such a set of candidate links could be established, for instance, as the top ranking genes from a genome-wide association study and their potential relationships to the node representing the phenotype being studied. Under the assumption of correlation between biological relatedness and graph proximity, ranking genes by their proximities to the phenotype enables extracting the most promising candidates for further study. As another example, a list of candidate links to be evaluated can result from a genome-wide protein interaction screen, where only a minority of identified putative interactions are biologically relevant [8].

In the following paragraphs, we will define and consider a number of node proximity measures suitable for weighted graphs such as Biomine. In the Results section we will evaluate the hypothesis that links can be predicted applying such measures on the data in Biomine.

#### Proximity measures

We consider four existing proximity measures: *probability of best path*[9], *network reliability*[28], *expected reliable distance*[29], and a weighted version of *rooted random walk with restart*[27]. Of these, the first three are specifically defined for probabilistic graphs, while the rooted random walk is normally used for unweighted graphs, but can be modified straightforwardly to take edge weights into account.

##### Probability of best path

Each edge *e* has a *probabilityp*(*e*) ∈[0,1]. Let *pathP* consist of edges $e_{1},\ldots ,e_{k}$. The path exists only if all of its edges exist, and correspondingly the probability of *P* is the product of the probabilities of its edges: *Pr*(*P*) = *p*(*e*_1_)·…·*p*(*e*_*k*_).

The simplest possible proximity measure for two nodes *s*,*t* ∈ *V* is the *probability of the best path*:

(1) $$p_{\mathrm{bp}}(s,t)=\underset{P\;\text{is a path from}\;s\;\text{to}\;t}{\max}\mathrm{Pr}(P).$$

An obvious potential shortcoming of this measure is that it does not take into account other paths between *s* and *t*.

##### Network reliability

To specify the next two, more complex proximity measures that are not restricted to considering the single best path, we first define a probabilistic graph model. Let *G* = (*V*,*E*,*p*) be a probabilistic graph, such as a subgraph of Biomine. *g* is a random realization of *G* if it is a non-probabilistic graph *g* = (*V*,*E*_*g*_) with nodes *V * and with edges sampled from *E* according to the probabilities *p*, i.e., each edge *e* ∈ *E* is selected to be an edge of *g* with probability *p*(*e*), independently of other edges. The probability of a given random realization *g* thus is

$$\mathrm{Pr}(g)=\underset{e\in E_{g}}{\prod }p(e)\underset{e\in E-E_{g}}{\prod }(1-p(e)).$$

The *Network reliability*, *p*_*r*_(*s*,*t*) between nodes *s* and *t* is defined as the probability that a randomly picked instance of *G* contains a path between *s* and *t*:

(2) $$p_{r}(s,t)=\underset{g|s\;\text{and}\;t\;\text{are connected in}\;g}{\sum }\mathrm{Pr}(g).$$

##### Expected reliable distance

Given a graph *g* sampled from *G*, we denote the shortest-path distance (measured as the number of edges) between *s* and *t* by *d*_*g*_(*s*,*t*).

The *expected-reliable-distance*[29] is now defined as the expected shortest-path distance in all instances *g* in which a path exists between *s* and *t*:

$$d_{\mathrm{ER}}(s,t)=\frac{1}{p_{r}(s,t)}\cdot \underset{g|s\;\text{and}\;t\;\text{are connected in}\;g}{\sum }\mathrm{Pr}(g)\cdot d_{g}(s,t).$$

The expected-reliable-distance reflects the expected proximity of nodes *s* and *t*, but does this on the condition that they are connected.

##### Random walk with restart

As a final probability measure, we consider a symmetric, weighted version of a standard random walk stationary distribution score with restarts[27]. We first define a directed version of the score. A random walk starts at a node *s*. It then iteratively moves to a random neighbor of the current node *x*, such that the probability of traversing edge *e* is proportional to the edge weight *p*(*e*). Additionally, there is a fixed probability *β* of returning to the initial node *s* at each step, instead of traversing an edge. Now, the directed version of the score, $d_{RW^{\prime }}(s,t)$ is defined simply as the stationary distribution probability of the walker being at node *t* after indefinitely many iterations. The final, symmetric version of the score is defined as the average of the corresponding directed scores:

(3) $$d_{\mathrm{RW}}(s,t)=\frac{d_{RW^{\prime }}(s,t)+d_{RW^{\prime }}(t,s)}{2}.$$

In practice, we compute the score by simulating a random walk from both nodes of the pair in turn, using 1,000,000 iterations, and counting the number of iterations where the walker is located in the other node.

## Results

We now test Biomine as a resource for biological link prediction, first with experiments predicting protein interactions and phenotypical relationships of genes. The main goal of the experiments in the first subsection is to demonstrate that the proposed approach of combining data from heterogeneous data sources into a single graph proximity measure is beneficial. Secondary goals are assessing the relative importances of different types of data and finding suitable weights for them, as well as evaluating the different node proximity measures defined in the Methods section. While the optimization of edge weights serves as a simple example of how to optimize parameters in Biomine, a thorough optimization of all the parameters is outside the scope of this paper. In the second subsection we will consider the specific application of prioritizing putative disease genes.

### Predicting future links

The aim in the first evaluation is to predict protein interactions and phenotypical relationships of genes. The tests in this section are carried out with two versions of the Biomine database: *old* from June 2007, and *current* from June 2010, such that the current version is used to validate predictions made using the old version. This prediction task is scientifically interesting and most challenging: can we predict which links will be discovered and added to the source databases? Predictions are performed by ranking node pairs by their proximity values, as defined by one of the general graph-based proximity functions defined in the previous section.

We consider two different link prediction settings:

1.Predict *protein interactions* that will be added to the Entrez Gene database [33] in the three-year period between the database versions.

2.Predict *pairs of genes that will be discovered to affect the same disease* during the three-year period. These are based on gene-phenotype associations reported in the Entrez Gene database, from the two genes to a shared phenotype. (At least one of them must be new.)

#### Evaluation methodology

Given a list of node pairs (i.e., potential links) and a proximity function *f *, we rank the given node pairs by *f * computed using the old version of the Biomine graph database. The hypothesis is that pairs ranking higher are more likely to be biologically related and thus to be directly linked in the current database version. The pairs that became linked are the *positive instances* while all pairs that remained unlinked are considered *negative instances* (although it is of course possible that they will actually become linked later on).

We measure the prediction performance using *ROC analysis*[40], a generic framework for analyzing and comparing classifiers. In our case, a proximity measure immediately gives a classifier: fix a cut-off value *q* ∈[0,1] for the proximity, then predict all pairs (*uv*) with *f*(*uv*) ≥ *q* to become linked and all other pairs to remain unlinked. In ROC analysis, two statistics are associated to each classifier: (1) the *true positive rate*, which in our case is the fraction of positive instances that have proximity at least *q*, and (2) the *false positive rate*, which is the fraction of negative instances with proximity at least *q*.

In the ROC framework, a proximity measure can conveniently be evaluated without fixing a single cut-off value *q*. A *ROC plot* is a two-dimensional curve that plots the true positive rate on the *y*-axis and the false positive rate on the *x*-axis. A single classifier (or in our case a single cut-off value *q*) corresponds to a single point in the ROC plot, and a ROC curve is obtained by considering all possible cut-off values *q*. Such a curve depicts the different tradeoffs between the benefits (true positives, *y*-axis) and costs (false positives, *x*-axis). In practice, ROC curves can be constructed by iterating the node pairs in ranked order and plotting the curve accordingly, with *q* being defined implicitly by the fraction of instances covered at any point.

The resulting ROC curves can be compared either visually, or by computing the area under ROC curve (*AUC*) as a composite statistic. AUC has an interpretation as the probability that a randomly chosen positive instance will be ranked above a randomly chosen negative instance [40]. We also evaluate the statistical significance of the difference in AUCs between two classifiers, using the statistical ROC analysis tool StAR[41].

We sample a set of positive and negative instances for the evaluations, as described below. In those experiments where we optimize parameters, we use separate samples for training (parameter optimization) and validation (evaluating the final performance). Both the training and validation sets are sampled in the same way, under the constraint that no node pair appears in both data sets.

#### Sampling of test data

We performed the experiments by sampling 500 positive and 500 negative instances for each of the two settings as follows.

##### Sampling of protein interactions

We first extracted the set of all protein interaction edges that exist only in the current database version. From these interactions, we randomly sampled 500 node pairs as the positive instances. A set of 500 negative instances was then sampled by randomly pairing nodes appearing in the set of positive instances, excluding any node pairs that have an interaction in the current database version. We picked the negative pairs in this manner to make the comparison as fair as possible, as a completely random selection of negative instances would most likely contain less researched proteins (with fewer links), unnecessarily making the prediction task easier.

##### Sampling of disease gene pairs

The set of potential positive instances was defined as those gene pairs that became linked by a path of two “Gene affects Phenotype” edges derived from the Entrez Gene database, going through any intermediate Phenotype node. First, all pairs of genes linked in the current database version were considered as candidates for positive instances. From these pairs, we removed ones that were already linked in the old database version by some path of length two consisting of any combination of following edge types:

“Gene affects Phenotype” (derived from Entrez Gene)

“Gene refers to Phenotype” (derived from OMIM or Entrez Gene)

“Phenotype refers to Gene” (derived from OMIM)

Of the remaining candidate pairs, we randomly sampled 500 positive instances to be used in the experiments. A set of 500 negative instances was then sampled by randomly pairing nodes appearing in this set of positive instances, such that all pairs linked (as described above) either in the old or new database version were excluded.

The sets of sampled positive and negative node pairs are available in Additional file 1.

#### Experimental results for link prediction

##### Comparison of proximity measures

We first performed a set of experiments to evaluate and compare the four proposed proximity measures: probability of best path, network reliability, expected-reliable-distance, and random walk with restarts. In this experiment we used uniform relevance *q* = 0.8 for all edge types, and the amount of degree penalty was set to *α* = 0.25. These are the parameter values that were found to perform best among a number of alternatives tested in our previous paper [9], for both the best path and network reliability proximity measures. For the random walk method, we set the restart probability *β* to 0.2 (roughly corresponding to the relevance value *q* = 0.8 used by the other, probabilistic distance measures).

**Figure 2:**
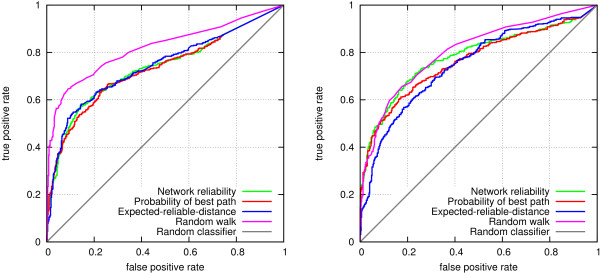
**ROC curves for link prediction accuracy using different node proximity measures.** Left: protein interactions. Right: disease genes.

Figure 2 shows prediction accuracies from this experiment as ROC curves. All measures give a prediction accuracy significantly better than would be obtained by a random classifier (the diagonal). In the protein interaction prediction task (left), differences between the three probabilistic methods are very small, but notably the random walk method achieves a clearly better accuracy compared to the other methods. The AUC scores obtained by the classifiers are 0.8200, 0.7425, 0.7362 and 0.7314 for random walk, expected reliable distance, two-terminal network reliability and probability of best path, respectively. The differences to random walk are statistically significant (p-values 0.0001, 0.0001 and <0.00001 for expected-reliable-distance, two-terminal network reliability, and probability of best path, respectively). The differences between the other classifiers were not statistically significant.

In the disease gene prediction setting (Figure 2, right), random walk is again most accurate overall. However, the difference to network reliability is non-significant (p-value 0.2596). More importantly, the two methods are comparable on the most relevant, leftmost part of the ROC curve. Probability of best path is equally accurate in that area, but slightly less accurate in other cases (p-value of AUC difference to random walk is 0.0466). The expected-reliable-distance is significantly inferior (the p-value of AUC difference to random walk is 0.0062).

We decided to use the random walk measure in the rest of the experiments of the paper, since it clearly outperformed the other methods in the protein interaction prediction task, and none of the other methods outperformed it in the disease gene prediction.

##### Choosing weights for different edge types

In the previous experiment, edges of all types were weighted uniformly. Suitably weighting different edge types is expected to improve prediction accuracy, as discussed above. Our next goal is to examine whether edges of different types can be weighted according to the prediction task to improve prediction accuracy.

For both of the considered prediction settings (protein interactions and disease gene pairs), we separately performed the following simple manual procedure to approximately optimize link prediction accuracy, as measured by AUC. In the optimization, we used one sample of 500 + 500 instances as a training set, and then evaluated performance with the resulting relevance coefficients using another sample of 500 + 500 instances as a validation set.

In preliminary experiments, it was observed that the most significant effect was obtained by adjusting the weight of the edge type “codes for”, that is, the links connecting genes to their protein products. The relevance *r*_*codes*_*for*_ was consequently first set to value 10 (increasing the coefficient beyond this value did not improve accuracy any more).

**Figure 3:**
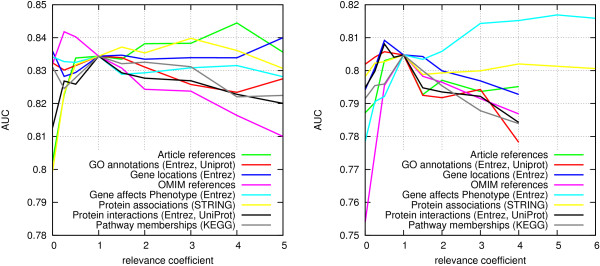
**Effect of edge type-specific relevance parameters on link prediction accuracy.** Left: protein interactions. Right: disease genes.

After setting *r*_*codes*_*for*_, we evaluated the individual effects of all other relevance parameters for the most abundant edge types in Biomine by varying them one at a time while keeping all the other parameters at the default value 1 (Figure 3; protein interaction setting on the left, disease gene pairs on the right). Based on this experiment, each relevance parameter was set to the value where the resulting AUC was maximized. This heuristic is simple and ignores any dependencies between edge types, so the result is not guaranteed to be optimal. On the other hand, the simplicity of the procedure helps avoid overfitting to the data. In the protein interaction setting, not all relevance parameters manifested any clear effect on accuracy, which is possibly partially due to the limited size of the training set (500+500 pairs), and partially to some edge types being less relevant than others for the task at hand. The relevances for these edge types were left at the default value of 1, as were the relevance coefficients for other, less common edge types not considered in this experiment.

The adjusted relevance values are listed in Table 3. The dashes denote cases where adjusting the relevance of the particular edge type did not have any noticeable effect on accuracy. Note that the values are not directly comparable, since larger relevances may result as a compensation for low informativeness: the most abundant edge types (such as article references and predicted protein associations) are likely to be least informative in our edge weighting model.

**Table 3:** Adjusted relevance parameters

**Edge type**	**Source databases**	**Relevance (interactions)**	**Relevance (disease genes)**
Article references	All	4.0	1.0
Gene locations	Entrez, OMIM	–	0.5
Gene *affects* Phenotype	Entrez	–	5.0
GO annotations	Entrez, Uniprot	1.0	0.5
OMIM references	OMIM	0.25	1.0
Protein associations	STRING	3.0	1.0
Protein interactions	Entrez, UniProt	1.0	0.5
Pathway memberships	KEGG	–	1.0
Gene-protein relations	KEGG	10.0	10.0

Value “–” indicates that adjusting the relevance of the edge type did not have a clear effect on accuracy.

**Figure 4:**
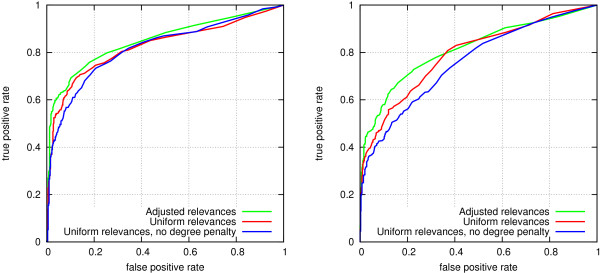
**ROC curves for link prediction accuracies using different edge weighting parameters.** Left: protein interactions. Right: disease genes.

To evaluate prediction performance with the adjusted relevance parameters, we next performed similar link prediction experiments using a separate validation set, with the following three sets of parameters: (1) uniform relevance and informativeness for all edge types (no degree penalty); (2) uniform relevance, degree penalty coefficient *α* = 0.25; (3) relevance parameters set to their adjusted values (Table 3), degree penalty coefficient *α* = 0.25. Again, separate experiments were performed for the two link prediction settings (Figure 4). The first version, using no degree penalty, is included in this experiment to demonstrate the usability of having a degree-based informativeness component in the edge weighting function, as all other experiments in the article have been performed using degree penalization.

Using adjusted relevances clearly improves accuracy in both settings: *AUC* = 0.824 vs. *AUC* = 0.849 (p-value 0.0005) for the protein interaction setting (Figure 4, left) and *AUC* = 0.792 vs. *AUC* = 0.814 (p-value 0.0002) for the disease gene setting (Figure 4, right). Moreover, the experiment shows that having a degree-based informativeness component in the edge weights is useful, especially in the disease gene prediction setting (*AUC* = 0.758 vs. *AUC* = 0.792, p-value < 0.0001), and to a lesser extent in the protein interaction setting (*AUC* = 0.818 vs. *AUC* = 0.824, p-value not significant).

Overall, prediction performance is clearly improved by suitably weighting the edges compared to using uniform weights: *AUC* = 0.758 vs. *AUC* = 0.814 (p-value <0.0001) in the disease gene setting and *AUC* = 0.818 vs. *AUC* = 0.849 (p-value <0.0001) in the protein interaction setting.

##### Accuracy of individual data sources

**Figure 5:**
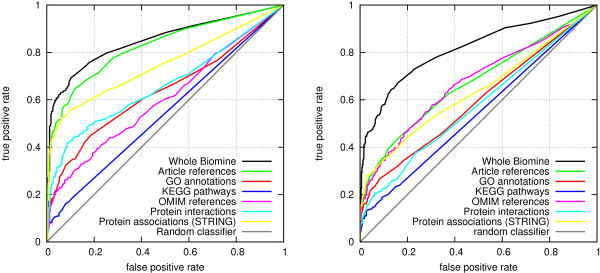
**ROC curves for link prediction accuracies using individual data sources.** Left: protein interactions. Right: disease genes.

Finally, we evaluate the value of integrating data from different heterogeneous sources, by comparing the link prediction accuracy obtained using data from each individual data source to accuracy obtained using all data in Biomine. These experiments are performed using the separate validation data set. Again, the test does not cover all edge types in Biomine, only the major ones: Gene ontology annotations, protein interactions from UniProt and Entrez Gene, participation of genes in pathways, protein associations from STRING, article references, and gene-gene and gene-phenotype references from OMIM. Figure 5 shows the prediction accuracy using each of these data types alone, as well as for the complete Biomine database (all data types combined) using the weights reported in Table 3.

The main observation from this experiment is that using all data in Biomine results in significantly better accuracy than using any single type of data alone. This is especially true for the disease gene prediction setting. Another observation is that the predictive power of different data types differs somewhat between the two link prediction tasks.

In the protein interaction setting, using only article references suffices to give a relatively good performance. Two main factors are likely to contribute to this result. First, links to articles are abundant (cf. Table 2). Second, they relate many different types of nodes and thereby contain richer information than any other individual data source. Note also the article references are actually derived from several data sources, although they are reported as a single data type in this experiment.

##### Summary of results for link prediction

We briefly summarize the experimental results from this section below. A more elaborate treatment is deferred to Discussion.

1.Biomine can be used to predict future links with high accuracy.

2.A random walk proximity measure performed best among the four tested node proximity measures.

3.Link prediction accuracy was improved by experimentally adjusting the weights of different edge types. The adjusted relevance coefficients generalized to separate validation sets of node pairs with good results.

4.Applying a degree penalty based on node degree improved prediction results.

5.Integration of multiple data types produced superior results over any individual type of data.

### Disease gene prediction

As a second example application, we next consider using Biomine for the refinement of results from genome-wide association studies, that is, identifying the actually relevant genes from the list of all genes showing statistical association to the disease. As discussed above, our approach is based on the assumption that genes proximal in the integrated graph of biological associations are more likely to be related to the same disease than a pair of more distant genes.

We formulate the task as a classification problem. Assume a set *S* of statistically disease-associated genes from an association study, where only genes in the subset *S*_*P*_ ⊂ *S* actually increase susceptibility to the disease, i.e., they are the *(true) positives*. The rest, *S*_*N*_ = *S* − *S*_*P*_, are *negatives* or false positives of the association study. Also assume a proximity measure *p*(·), as in the previous section. The task now is to predict *S*_*P*_ (and *S*_*N*_) by outputting an estimate ${\hat{S}}_{P}\subset S$.

We consider two alternative formulations of the problem:

**supervised classification** using only positive instances (see, e.g., George et al. [2] and Kohler et al. [16]). In this easier formulation we are given, in addition to *S*, a separate reference set *S*_*R*_ of genes already known to increase susceptibility to the disease. The predictions are then based on proximities between elements of *S* and *S*_*R*_.

**unsupervised classification** (see, e.g. Franke et al. [18]). In this “de Novo” version of the problem, we do not assume information about known disease genes. Instead, *S* is the only input. In this case, the predictions are based only on the mutual proximities of the genes in *S*.

In the supervised version of the problem, the idea is that among the statistically associated genes, those proximal to already known disease genes will be identified as the most promising candidates. In the unsupervised version, such existing information is not assumed. Instead, associated genes that are close to other associated genes will be considered as the most likely candidates.

#### Classifiers

We use the random walk with restart (Equation 3) as the proximity measure *p*(*s*,*t*) in these experiments, as it outperformed the other tested measures in the link prediction experiments above, and also performed consistently well in comparison to other methods in the disease gene prediction problem considered here (results not shown). Based on the pairwise proximity measure, we next define three alternative classifiers.

##### Supervised classifier

For the supervised version of the problem, we simply rank genes in *S* based on their average proximity to elements of the reference set *S*_*R*_:

(4) $$\mathrm{scor}e_{\mathrm{prox}}^{A}(s)=1/|S_{R}|\cdot \underset{t\in S_{R}}{\sum }p(s,t).$$

This definition is closely related, although not identical to the one used by Kohler at al. [16]. A binary classifier is obtained by setting a threshold *q*: ${\hat{S}}_{P}=\{s\in S:\mathrm{score}(s)\geq q\}$; ${\hat{S}}_{N}=\{s\in S:\mathrm{score}(s)<q\}$.

##### KNN classifier

In the unsupervised version of the problem, there is no fixed reference set; instead we just rank each gene by its proximity to *k* nearest elements of *S*:

(5) $$\mathrm{scor}e_{\mathrm{knn}}^{B}(s)=1/k\cdot \underset{\begin{array}{c} S^{\prime }\subset S-s,\\ |S^{\prime }|=k \end{array}}{\max}\underset{t\in S^{\prime }}{\sum }p(s,t).$$

This is motivated by the fact that random genes (false positives) are not likely to have many close neighbors in *S*; on the other hand, genes actually related to the disease are expected to be proximal to each other, and thus likely to be found in the set of *k* nearest neighbors. This definition can be seen as a generalization of the scoring scheme used by Franke et al. [18]). Again, a classifier can be obtained by simply thresholding, as for the *Supervised* classifier above. For the experiments of this section, we have used a fixed value of *k* = 4 for the number of neighbors. (This value was found to be optimal in preliminary experiments where the number of positive genes to be discovered was 5.)

##### Cluster-based classifier

We propose the following new method for the unsupervised version of the problem. Do not rank individual genes; instead find a single cluster ${\hat{S}}_{P}\subset S$ of genes that maximizes

(6) $$\mathrm{scor}e_{\mathrm{clus}}^{B}({\hat{S}}_{P})=\underset{\begin{array}{c} s,t\in {\hat{S}}_{P}\\ s\neq t \end{array}}{\sum }(p(s,t)-q).$$

Label the rest of the genes ${\hat{S}}_{N}=S-{\hat{S}}_{P}$ as negative. Here, *q* is a parameter governing how proximal a gene should be on average to the other members of the cluster to be considered positive. This definition may be best explained by considering the decision of whether to add a new gene *s* to some current estimate of ${\hat{S}}_{P}$. Adding a gene *s* increases the score if the average proximity of *s* to the genes already assigned to ${\hat{S}}_{P}$ is larger than the constant *q*. The definition is similar to the *maximum edge-weighted clique* problem [42], and also related to the outlier detection problem in clustering [43]; the difference to the latter one is that here only one cluster is sought, and searching the cluster and handling of outliers is done in a single integrated step.

A practical way of using the *Cluster-based* classifier is to vary the sensitivity parameter *q* in order to obtain a number of predicted sets of different sizes. Since the predicted sets are not monotone, that is, a smaller predicted set is not necessarily a subset of a larger prediction, there is not necessarily an immediately implied ranking of genes. However, as a rule, genes that appear in smaller predictions and more often can be considered more clustered.

To implement the *Cluster-based* classifier, in particular to find the ${\hat{S}}_{P}\subset S$ that maximizes Equation 6 for a given value of *q*, we use greedy search with multiple initializations, as follows. We maintain an estimate of ${\hat{S}}_{P}$. At each step of the algorithm, we test all possible moves of a single element between ${\hat{S}}_{P}$ and ${\hat{S}}_{N}$. There are thus |*S*| possible moves. Let *C* denote the current value of $\left({\hat{S}}_{P},{\hat{S}}_{N}\right)$ and let *C*_*s*_ be the candidate situation after moving *s* from its current set to the other set. For each possible move *s* ∈ *S*, we compute the improvement *Score*(*C*_*s*_)−*Score*(*C*) and choose the *s* that maximizes it. The algorithm is terminated when the score cannot be improved any more by moving any single element. We perform the above greedy algorithm for a number of different, randomly chosen initial estimates of ${\hat{S}}_{P}$, to reduce the problem of convergence to local optimum. In the experiments described next, we have used 100 restarts (increasing the number beyond this value did not yield any practical improvements in accuracy).

#### Test settings

A problem with systematic evaluation of disease gene prediction performance is that although results from several genome-scan experiments are publicly available (see e.g. [44]), the “correct” answer (genes actually affecting susceptibility to the disease) is not known. Therefore, instead of using actual genome-scan data, we evaluate the performance of the proposed methods by using artificial gene lists simulating lists of top-ranking genes from an association study, where the positive instances come from 110 already known disease gene families compiled by Kohler et al. [16]. These disease families have been constructed using information primarily from the OMIM database [38], augmented by domain knowledge and literature or database searches to find all genes clearly associated with each disease.

##### Generation of test cases

Each test case is a set *S* of genes, consisting of a fixed number of “positive” genes *S*_*P*_ from one of the 110 disease gene families, and a control set *S*_*N*_ of “negative” genes chosen at random from the other disease gene families. The control genes were chosen from the other disease families instead of all genes in the database to avoid bias caused by the fact that known disease genes have usually been studied more and thus have more edges in the database.

The original 110 families contain a total of 783 genes with 665 distinct genes, whereby the largest family contains 41 genes and the smallest only three genes. As a baseline setting, we consider prioritizing gene sets with |*S*_*P*_| = 5 and |*S*_*N*_| = 15, and thus |*S*| = 20 genes in total. We will then evaluate more challenging settings by varying these parameters. The number of genes in the disease gene families is a limiting factor in designing the experiments, and this choice means that only those 68 families that have at least 5 members can be used as positive instances in the experiments.

We performed experiments varying both the number of positive and negative instances, with |*S*_*P*_| ∈ {2,3,4,5} and |*S*_*N*_| ∈ {5,15,25,35,45}. For each combination (|*S*_*P*_|,|*S*_*N*_|), 100 test cases were generated, each containing |*S*_*P*_| genes sampled from a single disease gene family and |*S*_*N*_| genes sampled from among the other 109 disease gene families. As there were only 68 < 100 disease gene families with at least 5 members, 32 of the families are used twice for each set of tests with 5 positive genes. The generated test cases are available in Additional file 2.

Due to the limited number of genes available in the original disease gene families, we did not sample separate sets *S*_*R*_ to be used in the experiments with the supervised problem setting. Instead, we use the already sampled sets *S*_*P*_ to define *S*_*R*_, using the following leave-one-out cross-validation procedure: when computing the score defined by Equation 4 for each gene *p* ∈ *S*_*P*_, we use *S*_*P*_∖{*p*} as the reference set *S*_*R*_. To compensate for this, also the negative genes are similarly scored using |*S*_*P*_|−1 reference genes only, where a subset $S_{P}^{\prime }\subset S_{P}$ with $|S_{P}^{\prime }|=|S_{P}|-1$ is used to score each negative gene *n* ∈ *S*_*N*_ (it is ensured that each possible subset $S_{P}^{\prime }$ is used equally many times).

The results obtained using this approach are expected to be equivalent with results that would be obtained by using distinct sets *S*_*R*_ (with |*S*_*R*_| = |*S*_*P*_|−1), while enabling using a larger number of the original gene families for the experiments; if *S*_*P*_ and *S*_*R*_ were to be sampled separately, then only the 27 gene families that have at least 9 genes would be available for the experiments, which is only 39% of the families usable with the described cross-validation approach (the ones with at least 5 members).

##### Different versions of Biomine data

Some of the test cases are unrealistically easy to solve using Biomine, since it may contain edges that directly reflect knowledge about the disease gene families. For instance, direct textual references between genes belonging to the same disease family could have been derived from the OMIM database. To make the test settings here more realistic and challenging, we also carried out experiments where the most obvious sources of phenotype-related data were excluded. More specifically, we performed the experiments of this section using the following two alternative versions of the Biomine graph:

*Complete data* includes all data in Biomine.

*Reduced data* was obtained from the complete data by removing the following entities and links that most directly relate to the problem (and that were observed to have significant roles in the experiments of the previous section, cf. Figure 5): Although even after removing these data types there is “trivial” data left, the aim is that performing experiments with this more challenging subset of the data will reveal how the methods perform when there is less data on which to base the inferences.

Remove all phenotype nodes (and edges incident to those nodes). This avoids direct references to phenotypes.

Remove all edges derived from the OMIM database. This avoids references closely related to phenotypes.

Remove all edges derived from the STRING database. This removes predicted protein relationships, which may partly be based on data derived from OMIM.

##### Using ROC to evaluate classifiers

The *Supervised* and *KNN* classifiers can directly be evaluated in the ROC framework, and we again use both visualization of ROC curves and area under curve (AUC) as an overall score.

The *Cluster-based* classifier does not directly rank the genes but instead finds a partition into positive and negative instances. Any candidate cluster thus gives a point in the ROC space, but the clusters have to be computed separately for each value of *q*, unlike with methods that directly produce a ranking of genes. We compute a pseudo-ROC curve for the *Cluster-based* method by varying the sensitivity parameter *q* over a set of predefined thresholds. For each threshold, we perform experiments for all the 100 data sets. We then compute the true positive rate and false positive rate for each data set, and average these over all the data sets to obtain a composite point in the ROC space for each value of *q*. These points are then plotted to obtain the final ROC curve. (Unlike a standard ROC curve, the resulting curve is not guaranteed to be monotone, but deviations from monotonicity seem to be small in practice.)

##### Comparing results between the two versions of the ranking problem

The two problem settings, the supervised and unsupervised one, are not directly comparable since they are not really practical alternatives: the supervised method should be used whenever a reference set of known disease genes is available, since this helps in ranking; and when such a reference set is not available, there is no other option but to use an unsupervised method. Thus, a fair comparison is not straightforward to set up. Nevertheless, such a comparison between the methods can provide insight on how crucial having a pre-known reference set is for the prediction task.

We compare the classifiers in settings where each (positive) gene is scored using information from the same number of other positive genes by all methods. For example, when |*S*_*P*_| = 5, each gene within *S*_*P*_ is ranked using the 4 other positive genes by the *Supervised * classifier. For the *KNN* classifier, each positive gene is ranked using the 4 nearest neighbors (in the optimal case the 4 other positive genes), while in the *Cluster-based* method, each positive gene can potentially cluster with the 4 other positive genes.

#### Experimental results for disease gene prediction

##### Comparison of classifiers

First, we test the three proposed classifiers on the problem of identifying a set *S*_*P*_ of 5 disease genes among a set of 15 unrelated genes *S*_*N*_. 100 independent test cases of 20 genes are analyzed, as described above. The questions addressed by this setting are (1) how well disease genes can be prioritized using Biomine using any of the methods; (2) how much more difficult it is to prioritize disease genes without a reference set of pre-known disease genes; and (3) how well the cluster-based classifier (Equation 6) works compared to the simpler baseline classifier based on the *k* nearest neighbors (Equation 5).

**Figure 6:**
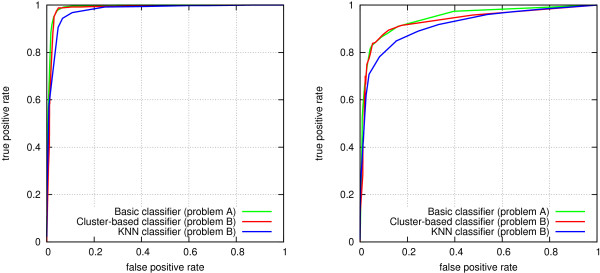
**Comparison of classifiers using ROC curves.** Left: protein interactions. Left: all data. Right: reduced data.

**Figure 7:**
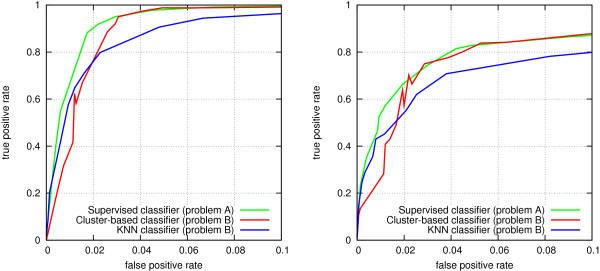
**Comparison of classifiers using ROC curves - FP rate *0−0.1*.** Left: all data. Right: reduced data.

Figure 6 reports the results from these experiments as a ROC curve averaged over the 100 independent test cases, using either complete data in Biomine (left) or the reduced data set (right). Figure 7 shows the first 10% of the same ROC curve. There are several observations from this experiment. First, using all data in Biomine (left), the true disease genes can be predicted with a rather high accuracy. Also in the more challenging case of reduced data (right), predictions can be made with reasonable accuracy. In both settings, the *Cluster-based* classifier obtains practically identical accuracy with the *Supervised* classifier for most of the ROC space, although it uses less prior information. It is also clearly superior to the *KNN* classifier. However, in the very beginning of the ROC curve (Figure 7) the cluster-based method does not perform well. This is most likely because that beginning of the curve corresponds to stringent (large) values of *q*, where only a part of the true positive genes are included in the cluster; here, the cluster-based method is not yet able to utilize information from all positive genes, a limitation which does not affect the *KNN* method.

##### Effect of increasing the number of negatives

To make the problem more realistic and challenging, we next evaluate how the accuracy of the proposed methods is affected when the amount of false positives in the set of genes to be prioritized is increased. For this experiment, we used the 5 sets of 100 test cases with |*S*_*N*_| ∈ {5,15,25,35,45}. As in the previous setting, the amount of positive genes |*S*_*P*_| is fixed to 5, giving positive-to-negative ratios from 1:1 to 1:9 and total number of genes to be ranked |*S*| between 10 and 50.

Again, we test the three proposed classifiers, with complete and reduced data separately. For the *Supervised* classifier, AUC should not be affected by the increasing number of negatives, as the ranking of each gene always occurs using a fixed reference set, irrespective of the number of negative genes. On the other hand, the more challenging unsupervised problem becomes more difficult when the amount of negatives is increased.

**Figure 8:**
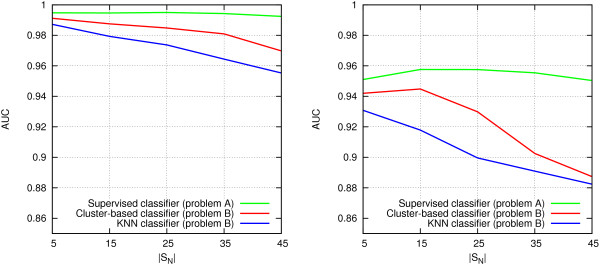
**Effect of increasing number of false positives on prediction accuracy.**|*S*_*P*_| = 5,|*S*_*N*_| ∈ {5,15,25,35,45}. Left: all data. Right: reduced data.

Figure 8 plots the AUC values obtained by each classifier as a function of the number of negative genes |*S*_*N*_|. Each point in the plot is an average AUC over the 100 independent test cases with a specific |*S*_*N*_|. As expected, AUC values for the *Supervised* classifier remain about the same in all settings. The accuracy of the other methods decreases quite steeply as the number of negatives increases. However, the cluster-based classifier is consistently superior over the *KNN* method, with a clear margin.

The results indicate that a reference set *S*_*R*_ is obviously useful, but if one is not available, relatively good predictions can still be obtained with the cluster-based method if the fraction of positive instances within the set of genes to be prioritized is sufficiently high.

##### Effect of decreasing the number of positives

**Figure 9:**
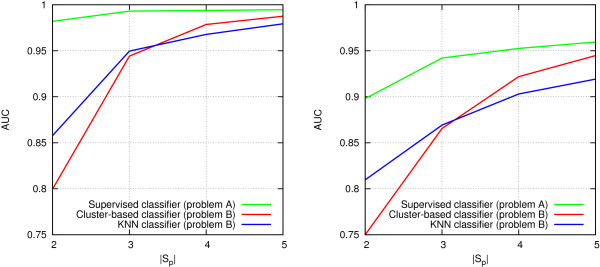
**Effect of decreasing number of true positives on prediction accuracy.**|*S*_*P*_| ∈ {2,3,4,5},|*S*_*N*_| = 15. Left: all data. Right: reduced data.

In the final experiment, we evaluate how decreasing the number of positives affects prioritization, by varying the number of positives |*S*_*P*_| between 2 and 5, while fixing the number of negatives to 15. Figure 9 plots the average AUC obtained by each classifier as a function of the number of positive genes |*S*_*P*_|. As expected, decreasing the number of positives has a dramatic effect on accuracy, especially in the unsupervised version of the problem, but also in the supervised version. Notably, the clustering-based method does not work well with the smallest values of |*S*_*P*_|, and is outperformed by the *KNN* method in these settings. Based on this experiment, it appears that at least 4 positive genes are required in the set to be prioritized in order to benefit from the cluster-based approach.

##### Summary of results for disease gene prediction

The results of this section can be summarized as follows.

1.The Biomine data and node proximity measures can be used to obtain a high accuracy in identifying actual disease genes from a putative set of genes. The best results are obtained, if a reference set *S*_*R*_ of known causal genes is available.

2.With a sufficient true disease gene density in the candidate list, the cluster-based method (Equation 6) performed almost as well. This is an interesting result, because the clustering method does not require a pre-known reference set. However, the cluster-based approach did not work well when the number of positive genes within the candidate list was small; in our experiments, at least 4 positive genes were needed for the cluster-based approach to be beneficial.

## Discussion

We first considered a number of node proximity measures as the basic element of link prediction. In these experiments, a random-walk based proximity measure was found to perform best. This result strongly contrasts results from a recent study comparing similar proximity measures on probabilistic graphs [29], where a method based on expected shortest path distance performed best, followed by probability of best path, network reliability, and finally random walk, with clear margins. The most striking difference is that in their link prediction experiments, random walk performed only slightly better than a random guess. We hypothesize that the main reason is the difference in the edge weighting schemes, which may be more suitable for some methods than for others. One difference in weighting is that the graphs of Potamias et al. contain more edges with probabilities close to 1.0, whereas in our scheme the types of edges and degrees of nodes have a larger effect on edge weights, and the resulting distribution of edge weights is more uniform.

Our empirical results show that the Biomine approach has strong statistical prediction power (see, e.g., Figure 5). However, the prediction accuracy is likely not sufficient for predicting arbitrary links within Biomine, as there are relatively few true positives among a huge number of potential links. Consider the current statistics of Biomine: the number of node pairs is of the order of 10^11^, while the current number of edges is of the order of 10^7^. For the sake of example, assume that the number of true positive links is 10 times larger than the number of current edges, i.e., 10^8^. The fraction of true positives among all potential links would be 10^8^/10^11^, i.e., one positive instance for every 1,000 negative ones.

Now, assume a true positive rate of 0.1 and a false positive rate of 0.0001, similar to experimental results in Figure 5. We are then 1,000 times more likely to classify positively a true positive than a true negative. Incidentally, this ratio is identical to the ratio of the negative and positive instances assumed above. In other words, the predicted positives would be expected to contain an equal amount of true and false positives. If one produced predictions for the whole Biomine, there would with these parameters be about 10^7^ true and false positives—clearly too much for any practical use.

In practical applications, such as analysis of protein interaction measurements or disease gene ranking, the set of potential links to predict is limited to a predefined set of candidate links that is already enriched with positive instances. This also means that although the proximity measure itself does not take into account the type of links to be predicted, the set of candidates is already chosen in such a way that the edge type is implicitly defined.

We next discuss our two test settings, protein interaction prediction and gene prioritization, and their results.

The protein interaction prediction experiments have been carried out with respect to new links introduced to the source databases between 2007 and 2010, with the above-mentioned good results. An interesting question is if and how much the methods are biased to making predictions in active areas of research. Existing information in the source databases reflects past and current research topics and hypotheses, and these may well correlate with future research and discoveries. A topic for future study is to investigate if certain types of links are easier to predict than others.

Use of Biomine in disease gene ranking enables identifying, from among a number of putative candidate genes, the ones that appear most plausible based on the data contained in the source databases. This approach is expected to work best in cases where several functionally related genes contribute to the disease, and knowledge about functions of the genes is already present in the source databases. Obviously, less studied genes with little or no functional annotations cannot be identified in this way.

We considered two versions of the gene ranking problem: one where genes are ranked based on their proximities to an already known reference set of disease-related genes (supervised setting), and another where ranking is based on the mutual proximities of the putative genes (unsupervised setting). Both formulations have already been considered in previous work[16,18]. The methods of producing data and computing proximities are different, however. Kohler et al. [16] only use a single type of edge (protein associations) while Franke et al. [18] collapse information from several data sources into a single type of edge. Neither of these approaches considers edge weights. In contrast, we retain the original edge types and construct a heterogeneous, weighted network. An additional difference is that the approach of Franke et al. is directly aimed at linkage studies where genes within continuous susceptibility intervals are examined, whereas we consider cases where the genes may be spread over the whole genome.

Powerful methods for disease gene prediction have been proposed by Hwang and Kuang [20] and Vanunu et al. [17]. They assume availability of three specific types of links (similarities between diseases, links from diseases to proteins, and protein interactions) and these are used in specific ways. In contrast, we do not make such assumptions about edge types: the link prediction methods used in this study are purely based on node proximities. While the approaches of Hwang, Kuang and Vanunu et al. can take better advantage of specific information, the methods used in this paper are more flexible and can also utilize unanticipated types of links.

Unlike the methods considered here, Wang et al. [21] and Chasman [22] do not use graph data, but instead perform joint testing of disease association within predefined sets of related genes (pathways and functional categories). In contrast, the methods applied in this paper are not limited to detecting association only within such predefined sets.

To sum up, the following combination of factors distinguishes our work from previous work on utilizing protein networks for disease gene prioritization:

use of weighted edges, with weights based on combining information from the type of edges, node degrees and weights in the original databases,

use of a heterogeneous graph, and

the novel single-cluster clustering formulation.

In this paper, the evaluation of Biomine has been carried out quantitatively, using numerical measures of prediction accuracy. Such measures are directly motivated by the prediction tasks considered in this study. An important, complementary application of Biomine is visualizing relationships between entities of the biological graph (cf. Figure 1), enabling the basis of predictions to be shown to the user for subjective analysis and verification. Finding connections previously unknown to the user may help understand biological mechanisms and produce new biological hypotheses.

Consider, for instance, the top ranking genes from a gene mapping study, or a gene that by some other evidence might be related to the phenotype under study. A subgraph that connects them [45] can be used to show the concrete chains of annotations that link the genes to the phenotype. Such use of Biomine is remotely similar to search engines: enter a number of query entities, and Biomine will search for chains and networks of entities that summarize the known relationships between the query entities. This search functionality is available in the Biomine web site http://biomine.cs.helsinki.fi.

## Conclusions

We presented Biomine, a system that integrates data from a number of heterogeneous sources into a single, graph-structured index. The experimental results indicate that Biomine enables performing useful prediction tasks, such as prediction of new links and ranking of putative disease genes.

Based on the experiments, a number of components contribute to the success.

1.**The Biomine database and data model**: both *integration of multiple types of data from several data sources*, as well as *use of weights on edges* were important factors.

2.**Suitable methods and measures for prediction**: *random walk with restart* worked best as a link prediction measure; for candidate gene ranking, slightly more complex *supervised or unsupervised prediction methods* (using random walk as a proximity measure) can be used depending on the availability of a reference set of known disease genes.

3.**Enriched data for the prediction problem**: Biomine has, together with the above-mentioned methods, statistically strong predictive power. However, for practical use in prediction or ranking tasks, a *selected set of candidate hypotheses* should be available. This is the case, for instance, in analysis of candidate genes from a genome-wide association study.

Our main motivation comes from gene mapping, in particular from analyzing and visualizing relations of candidate genes to the phenotype under study. In our experiments, Biomine had a high accuracy in identifying actual disease genes from a putative set of genes using a simple ranking scheme based on an already known reference set of disease genes. Experiments using a novel clustering-based method demonstrated that putative disease genes can also be ranked without an already established reference set, if the number and density of true disease genes is sufficient among the candidates. An interesting future research topic in this area is a semi-supervised setting where information both from a reference set of disease genes and from mutual proximities of the candidate genes is used. This might be useful especially in cases where only a small number of reference genes is available for the disease under study.

The current version of the Biomine database contains 1.1 million entities and 8.1 million relations between them, with focus on human genetics. The index can be queried using a public web interface on the Biomine web page. and results are visualized graphically. While gene mapping has been the motivating application, we believe that Biomine has applications in many other biomedical problems that benefit from integration of data and from the ability to estimate proximities of biological entities.

Biomine in its current form has a number of practical limitations. It only covers part of the available data, with focus on human genetics. Keeping the database updated and extending it requires resources. The database could be improved in a number of ways, including automatic learning of relevance coefficients for different edge types, or better semantic use of taxonomies such as Gene Ontology. Discovery of statistical relations between entities would be an interesting addition, for instance gene set enrichment analysis [46]. Naturally, future work will include applications of Biomine to specific biomedical problems.

## Competing interests

The authors declare that they have no competing interests.

## Authors’ contributions

LE designed and implemented a major part of the system, conceived the methods introduced in the paper, designed and carried out the experiments, and drafted the manuscript. HT participated in the design of the system and the study, and in drafting the manuscript. Both authors read and approved the final manuscript.

## Supplementary Material

Additional file 1 Gene pairs used in link prediction experiments Click here for file

Additional file 2 Gene sets used in disease gene prediction experiments Click here for file
